# Supplementary material for: Massive mutagenesis reveals an incomplete amyloid motif in Bri2 that turns amyloidogenic upon C-terminal extension
Source: Proc Natl Acad Sci U S A. 2025 May 2;122(18):e2415521122. doi: 10.1073/pnas.2415521122 (PMC12067230; doi:10.1073/pnas.2415521122)
Supplement: Supplementary file 1 — Appendix 01 (PDF) [file pnas.2415521122.sapp.pdf]

SI appendix

Extended Methods

Individual variant testing

Selected variants, from ADan and ABri libraries, for individual testing were obtained by PCR linearisation (Q5 high-fidelity DNA polymerase, NEB) with mutagenic primers (primers MM_68-85, Supplementary Data 2) or by ultramer amplification and Gibson assembly with the linearized plasmid for the sequences coming from the random libraries (primers MM_86-91). PCR products were treated with Dpn1 overnight and transformed in DH5α competent *E. coli*. Plasmids were purified by miniprep (QIAprep Miniprep Kit, Qiagen) and transformed into yeast cells. All mutated plasmids were verified by Sanger sequencing. For measuring growth in selective conditions, yeast cells expressing individual variants were grown overnight in a plasmid selection medium (-URA 2% glucose). They were then diluted to OD 0.1 in protein induction medium (-URA 2% glucose 100 μM Cu_2SO_4) and grown for 24 h. Cells were plated on -URA (control) and -ADE-URA (selection) plates in three independent replicates and allowed to grow for 7 days at 30 °C. Adenine growth was calculated as the percentage of colonies in -ADE-URA relative to colonies in -URA.

For individual growth rate measurements, yeast cells expressing individual variants were grown overnight in plasmid selection medium (-URA 2% glucose) and diluted to OD 0.2 until exponential. They were then diluted again to OD 0.1 in non-inducing (-URA 2% glucose) and inducing (-URA 2% glucose 100 μM Cu_2SO_4) protein expression mediums. Cell growth was measured at 30 °C for >48 h at 10 min intervals in a microplate reader (Spark, Tecan) in three biological replicates. Growth rates were calculated using the GrowthCurver package in R.

Thioflavin T Binding Assay

Synthethic purified peptides were purchased from Bachem and Genescript as TFA salts. The lyophilized peptides were resuspended with trifluoroacetic acid (TFA, Sigma) to 1 mg/ml concentration, sonicated for 30 s in ice, frozen for 10 min in an ethanol-dry ice bath and lyophilized overnight. The lyophilized products were resuspended in hexafluoro-2-propanol (HFIP, Sigma) to 1 mg/ml concentration, incubated for 10 min in ice and split in 50 μg aliquots. HFIP was removed by speed-vacuum and aliquots were stored at -80ºC. For the ThT kinetic, the HFIP treated peptides were resuspended in NaP 50 mM pH 7.4 buffer with 20 μM Thioflavin T (ThT). ThT fluorescence was measured reading at 480 nm (excitation at 440 nm) every 5 minutes with no shaking at 29ºC (Reader Infinite, Tecan). Peptide concentration was calculated by acid hydrolysis and amino acid analysis by the Separative Techniques Unit of the Cientific and Technological Centers of the University of Barcelona CCiTUB.

Spotting nucleation assay

GT409 [psi-pin-] and GT159 [psi-PIN+][15] were transformed with plasmids expressing SupN fused to Bri2, ABri, ABri A7P, ABri R24C, ADan, ADan K14V and ADan L20R. For measuring growth in selective conditions, yeast cells expressing individual variants were grown overnight in a plasmid selection medium (-URA 2% glucose). They were then diluted to OD 0.1 in protein induction medium (-URA 2% glucose 100 μM Cu_2SO_4) and grown for 24 h. 20 millions of cells

(OD~1) were used as the starting concentration (dilution 1:1) and serial dilutions 1:10 were carried out to reach a 1:10,000 dilution. 3 μl of each dilution were plated on -URA (control) and -ADE-URA (selection) plates and allowed to grow for 7 days at 30 °C. Relative growth was calculated as the percentage of colonies in -ADE-URA relative to colonies in -URA.

Supplementary Table 1. SNVs in BRI2 reported in GnomAD database. HGVS: Human Genome Variant Society nomenclature. AF: Allelic Frequency. NS: Nucleation Score.

ID	HGSVc	AF	HGVS ITM2B	HGVS ADan	NS ADan	Category (FDR=0.1) ADan	HGVS ABri	NS ABri	Category (FDR=0.1) ABri
rs1425712363	c.730G>A	3.99e-6	p.Glu244Lys	p.Glu1Lys	-1.20	NS-	p.Glu1Lys	-	-
rs1302882644	c.737G>A	3.99e-6	p.Ser246Asn	p.Ser3Asn	0.0448	WT-like	p.Ser3Asn	-	-
rs1365902742	c.743G>A	3.99e-6	p.Cys248Tyr	p.Cys5Tyr	-0.00901	WT-like	p.Cys5Tyr	-	-
rs765077508	c.747C>G	3.99e-6	p.Phe249Leu	p.Phe6Leu	-0.500	NS-	p.Phe6Leu	-0.109	WT-like
rs578006622	c.748G>T	4.38e-5	p.Ala250Ser	p.Ala7Ser	0.0708	WT-like	p.Ala7Ser	0.962	NS+
rs578006622	c.748G>A	7.97e-6	p.Ala250Thr	p.Ala7Thr	-0.0425	WT-like	p.Ala7Thr	-	-
rs139394220	c.751A>G	1.24e-4	p.Ile251Val	p.Ile8Val	0.0841	WT-like	p.Ile8Val	1.31	NS+
rs1272422512	c.755G>A	1.20e-5	p.Arg252Gln	p.Arg9Gln	0.474	NS+	p.Arg9Gln	-	-
rs1380885043	c.769A>G	6.37e-5	p.Lys257Glu	p.Lys14Glu	-0.00624	WT-like	p.Lys14Glu	-	-
rs1290822136	c.776C>T	7.97e-6	p.Ala259Val	p.Ala16Val	-0.233	NS-	p.Ala16Val	-	-
rs373968526	c.778G>A	1.59e-5	p.Val260Met	p.Val17Met	-1.07	NS-	p.Val17Met	-	-
rs1428007068	c.787T>G	3.99e-6	p.Leu263Val	p.Leu20Val	-1.46	NS-	p.Leu20Val	-	-
rs747798420	c.788T>C	7.97e-6	p.Leu263Ser	p.Leu20Ser	-3.53	NS-	p.Leu20Ser	1.84	NS+
rs771676204	c.790A>G	3.99e-6	p.Ile264Val	p.Ile21Val	-0.0338	WT-like	p.Ile21Val	0.211	WT-like
rs772731795	c.*3A>G	3.99e-6	-	p.Ser29Gly	-0.160	NS-	p.Thr25Thr	-	-
rs1163491396	c.*8A>G	3.99e-6	-	p.Gln30Gln	-	-	p.Lys27Arg	0.343	WT-like
rs760120359	c.*11A>G	1.20e-5	-	p.Glu31Glu	-	-	p.Lys28Arg	0.873	NS+
rs1455609180	c.*13A>C	4.00e-6	-	p.Lys32Thr	0.234	WT-like	p.Asn29His	-	-
rs770862229	c.*15C>T	4.00e-6	-	p.His33Tyr	-0.228	WT-like	p.Asn29Asn	-	-
rs776617945	c.*18T>C	4.00e-6	-	p. Tyr34His	0.0246	WT-like	p.Ile30Ile	-	-
rs759493813	c.*20T>C	7.10e-6	-	p. Tyr34Tyr	-	-	p.Ile31Thr	-	-

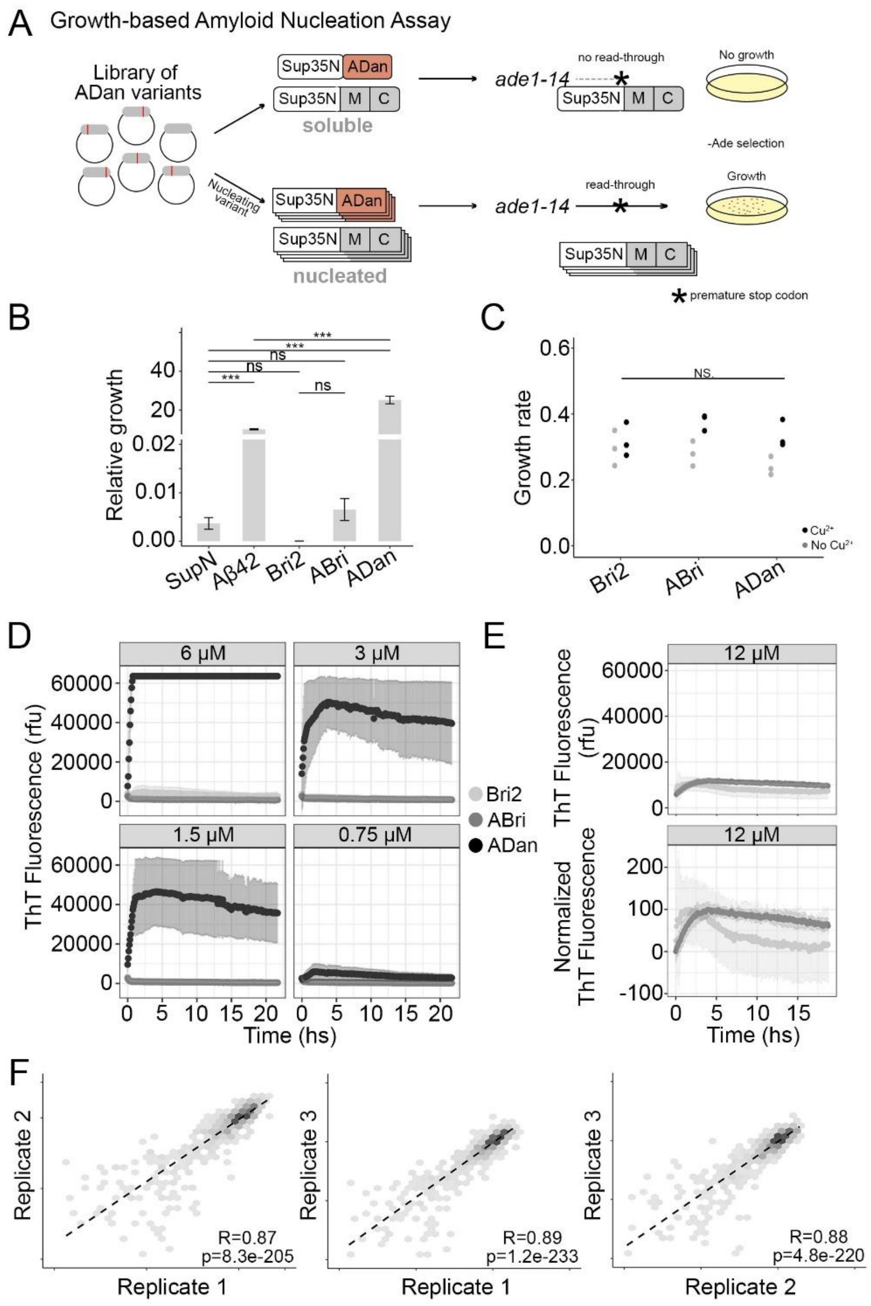

Supplementary Figure 1. ADan and ABri nucleation. A. Schematics of the reporter assay. **B.** Individual testing of SupN (negative control), Aβ42 (positive control), Bri2, ABri and ADan in the reporter assay. Relative growth of cells expressing each sequence, calculated as the number of colonies growing in the selective conditions of the amyloid nucleation assay (-URA, –Ade) over colonies growing in the absence of selection (-URA). Error bars indicate the standard deviation of the replicates (n=3). One-way ANOVA with Tukey's post-hoc test. $p<0.05$: *; $p<0.01$: **; $p<0.001$: ***. **C.** Individually measured growth rates for Bri2, ABri and ADan, in non-inducing (no Cu^{2+}) and inducing (Cu^{2+}) protein expression conditions (n=3 biological replicates/variant). One-way ANOVA with Dunnett's multiple comparisons test against Bri2 Cu^{2+}. **D-E.** The aggregation of Bri2, ABri and ADan peptides was followed using a continuous ThT binding assay. The concentration of the peptides is detailed in the upper part of each panel. Normalized ThT fluorescence was calculated relative to each trace. Error bars indicate the standard deviation of the replicates (n=3). **F.** Correlation of nucleation scores

between biological replicates for variants in the ADan library. Pearson correlation coefficient and p-value are indicated.

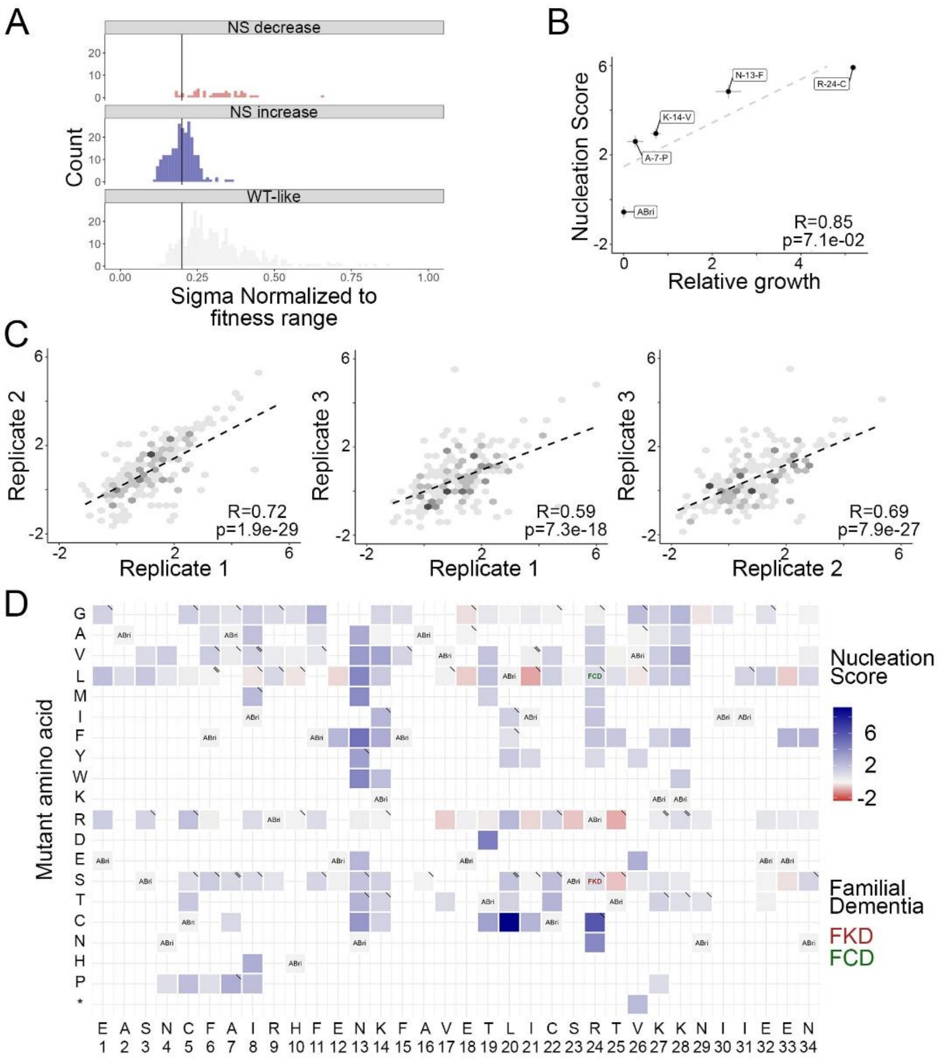

Supplementary Figure 2. Deep mutagenesis of ABri. A. Normalized sigma distribution for variants increasing, decreasing or not affecting nucleation at FDR=0.1. Vertical line indicates threshold value for considering low sigma. **B.** Correlation of nucleation scores obtained from selection and deep sequencing with relative growth of individual variants in selective conditions (n=5). Vertical and horizontal error bars indicate estimated sigma errors and standard deviation of the experiments (n=3), respectively. Pearson correlation coefficient and p-values are indicated. **C.** Correlation of nucleation scores between biological replicates for variants in the ABri library. Pearson correlation coefficient and p-value are indicated. **D.** Heatmap of nucleation scores for ABri single amino acid substitutions. The WT amino acid and position are indicated in the x-axis and the mutant amino acid is indicated in the y-axis. Synonymous variants are indicated as “ABri”, missense variants due to SNVs are indicated with “\” and SNVs present in GnomAD with a “\\” in the upper right corner of the cell. Mutation to stop codons are indicated with an “*”. Missing variants have been excluded on the basis of poor sequencing quality and low normalized sigma values (see Methods).

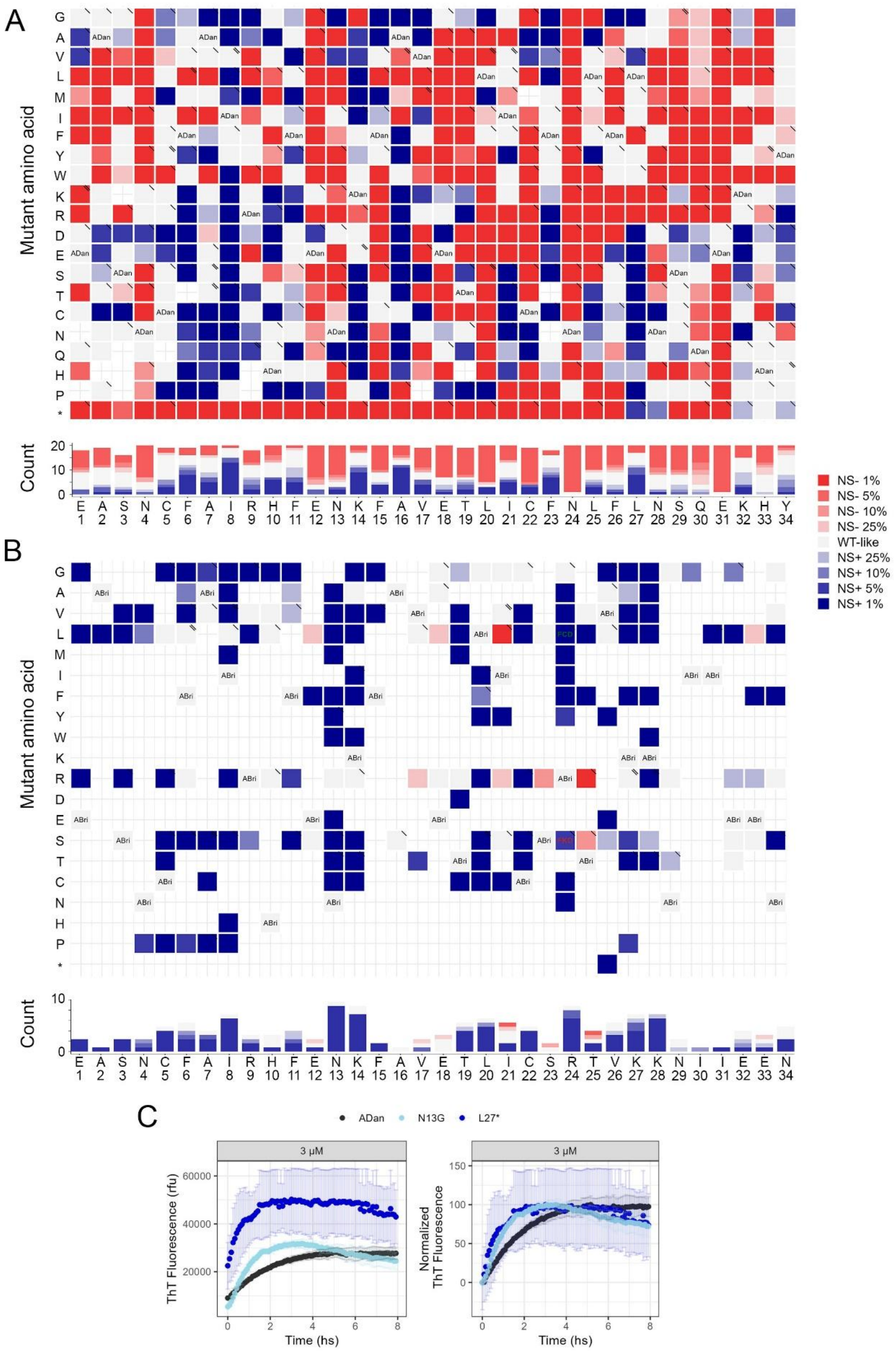

Supplementary Figure 3. Mutational effects of single AA substitutions. Heatmap of FDR categories for the effects of single amino acid substitutions. The WT amino acid and position are indicated in the x-axis and the mutant amino acid is indicated in the y-axis. Synonymous mutants are indicated with “ADan” (**A**) or “ABri” (**C**). Missense variants due to SNVs present

in GnomAD are indicated with “\” in the upper right corner of the cell. Mutation to stop codons are indicated with an “*”. The number of variants increasing or decreasing nucleation at different FDRs per position are indicated as stack bars at the bottom. **B.** The aggregation of ADan, ADan L27* and ADan N13G peptides was followed using a continuous ThT binding assay. The concentration of the peptides is detailed in the upper part of each panel. Normalized ThT fluorescence was calculated relative to each trace. Error bars indicate the standard deviation of the replicates (n=3).

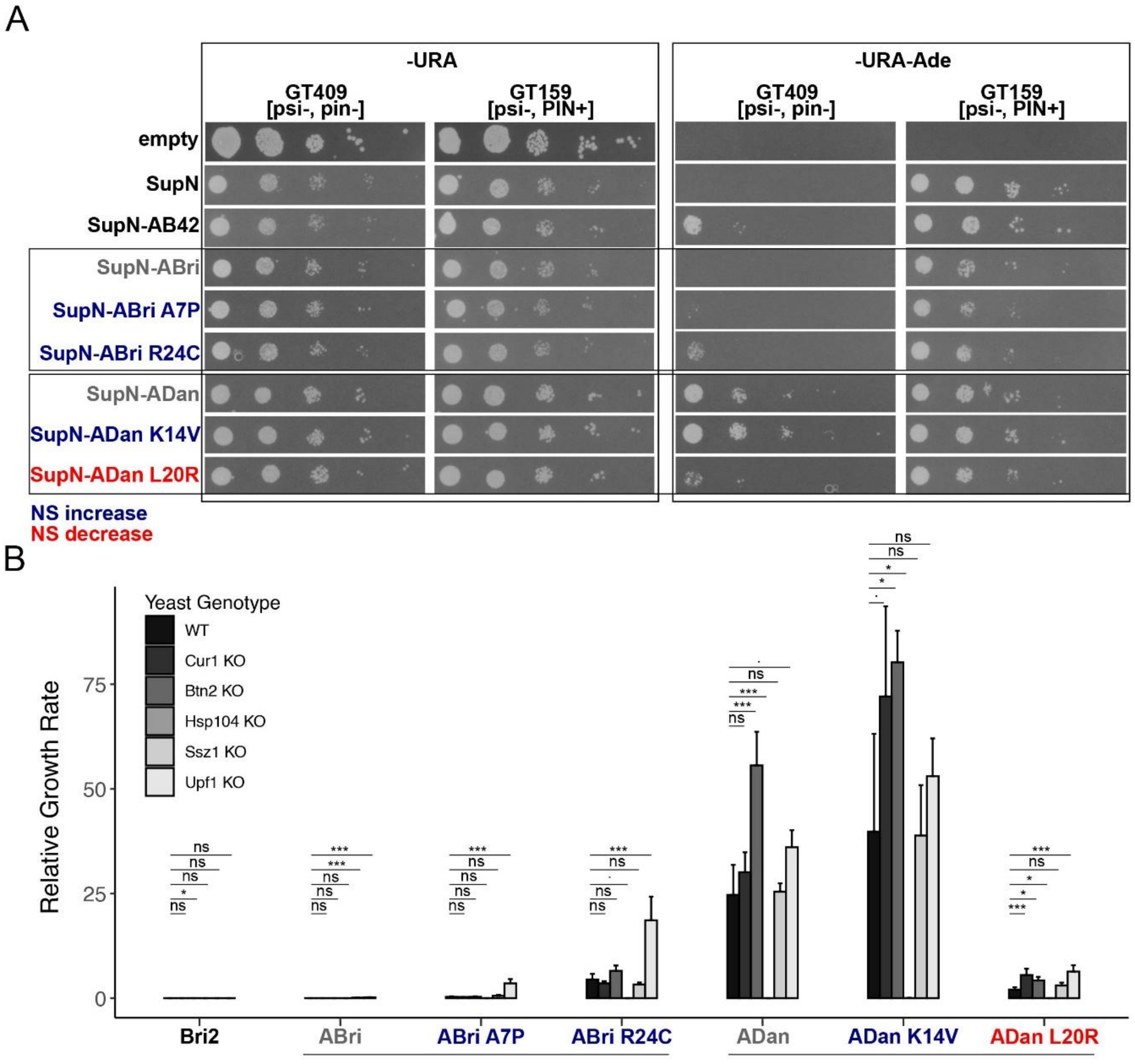

Supplementary Figure 4. Measuring selective growth in PIN+ and anti-prion KO backgrounds. A. [PSI+] induction by chimeric constructs in PIN+ background. Left panel: Growth control in non-selective conditions for protein aggregation. Right Panel: Growth in selective conditions for protein aggregation. [PSI+] formation (i.e. protein aggregation) is detected in selective conditions (-URA, -Ade) after induction (-URA, 100 Cu^{2+}). **B.** Relative growth of cells expressing SupN fused to Bri2, ABri, ABri A7P, ABri R24C, ADan, ADan K14V or ADan L20R in different genetic backgrounds where anti-prion systems (*cur1, btn2, hsp104, ssz1* and *upf1*) were knocked-out. *Hsp104* KO was used as a control for inhibition of prion fragmentation and propagation. Relative growth calculated as the number of colonies growing in the selective conditions of the amyloid nucleation assay (-URA, –Ade) over colonies growing

in the absence of selection (-URA). Error bars indicate the standard deviation of the experiments (n=3). One-way ANOVA with Dunnett's multiple comparisons test against wt background. $p<0.1$: ·; $p<0.05$: *; $p<0.01$: **; $p<0.001$: ***.

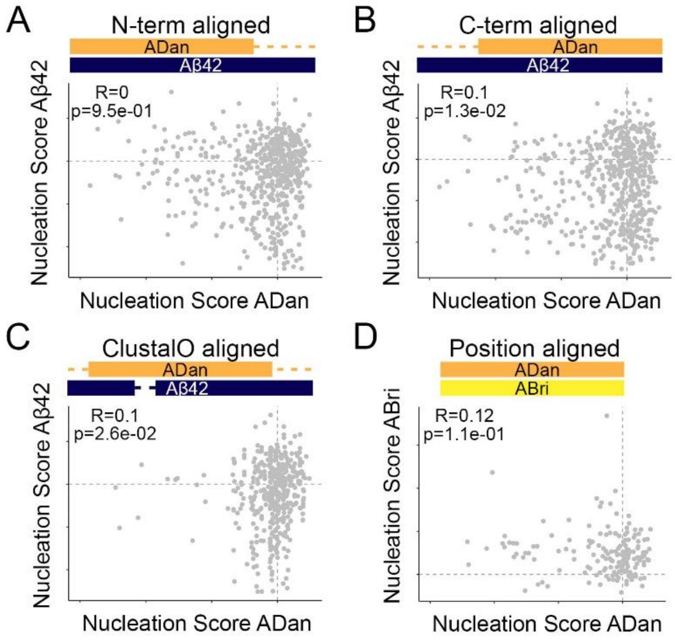

Supplementary Figure 5. Comparing the mutational effects of single amino acid variants in ADan, Aβ42 and ABri libraries. Correlation of nucleation scores between ADan and Aβ42 aligned at N-term (**A**), at C-term (**B**) and with ClustalO (**C**), and between ADan and ABri (**D**).

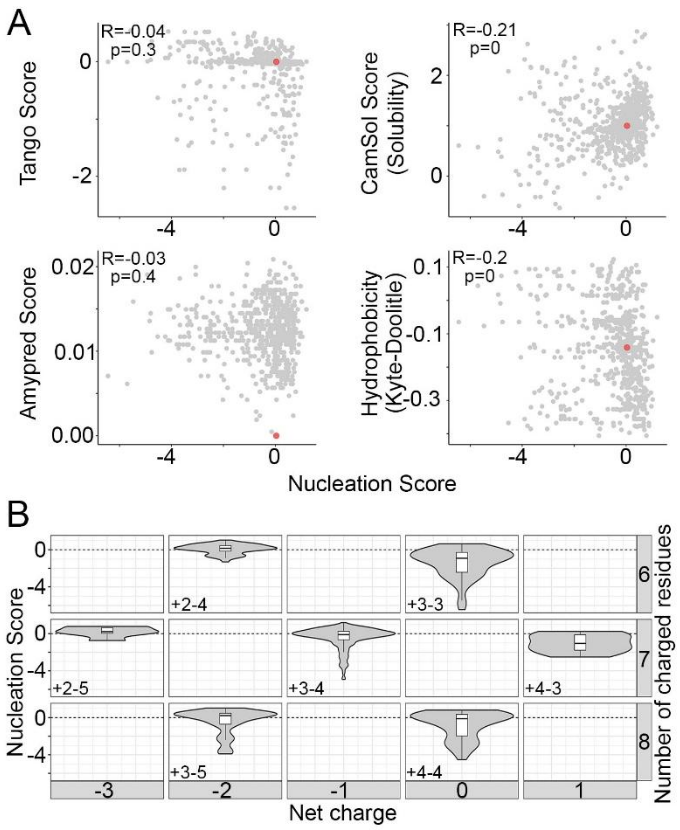

Supplementary Figure 6. Physicochemical features partially explain nucleation. A. ADan nucleation scores correlation with amyloid propensity predictors (Tango and Amypred), solubility predictors (CamSol) and Kyte-Doolittle hydrophobicity score. Red dots indicate the values for the WT sequence. Pearson correlation coefficient and p-values are indicated. **B.** Nucleation score distributions arranged by the number of charged residues (y-axis) and the total net charge (x-axis) in ADan. Numbers inside each cell indicate the number of positive and negative residues. The horizontal line indicates WT nucleation score (0).

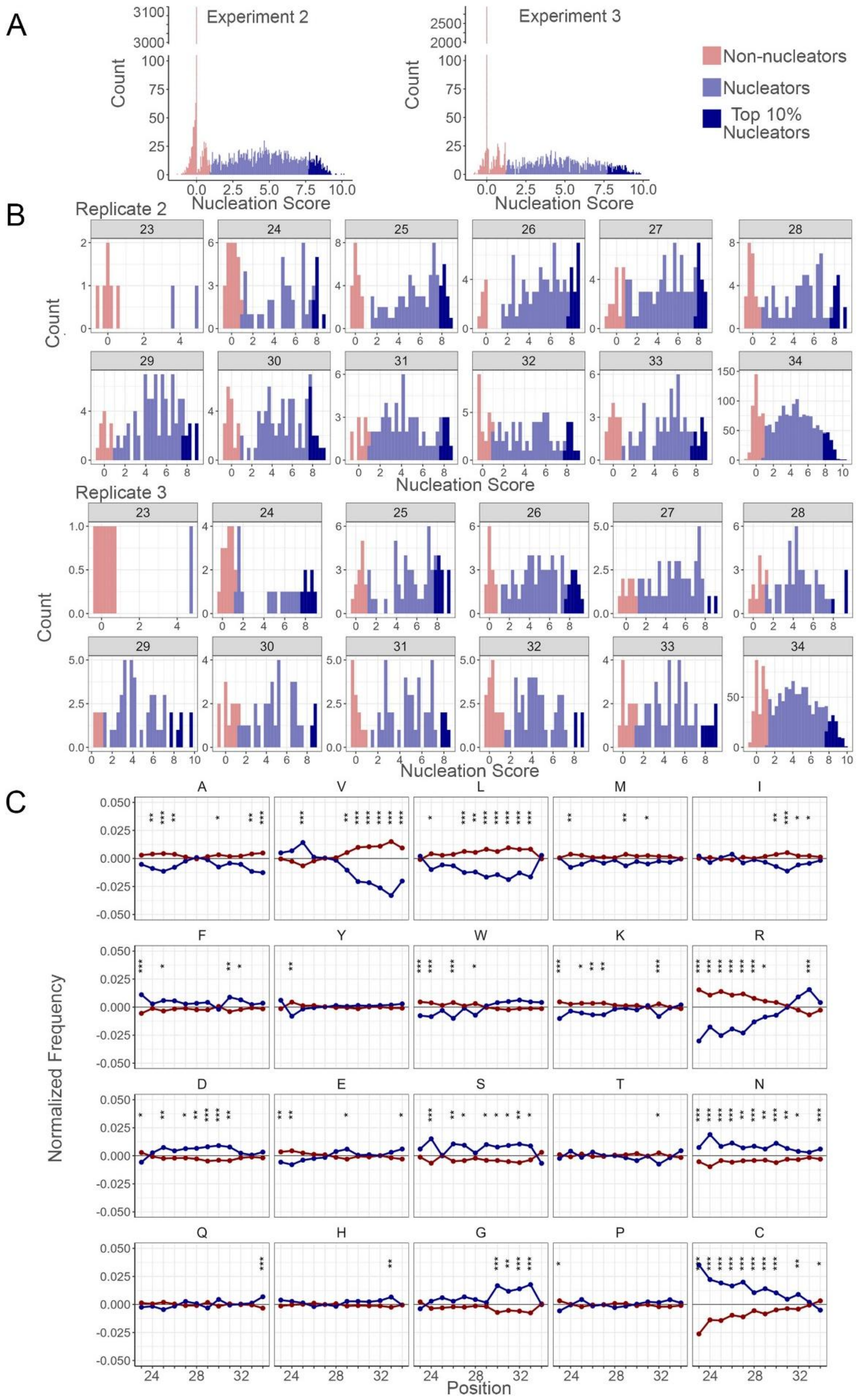

Supplementary Figure 7. Bri2 random extensions. A. Distribution of nucleation scores for random sequences of length 34 for replicates 2 and 3. Variants are classified as non-nucleators (red), nucleators (blue) or top 10% nucleators (dark blue). **B.** Nucleation scores distribution of random sequences present in replicate 2 and 3 (top and bottom, respectively).

The length of the peptide is indicated inside the grey box of each plot. Variants are classified as non-nucleators (red), nucleators (blue) or top 10% nucleators (dark blue). **C.** The position-specific differences in amino acid frequencies of sequences 34 amino acid long, across nucleating and non-nucleating sequences. Asterisks indicate marginal p-value (chi-square test). p<0.05: *; p<0.01: **; p<0.001: ***.

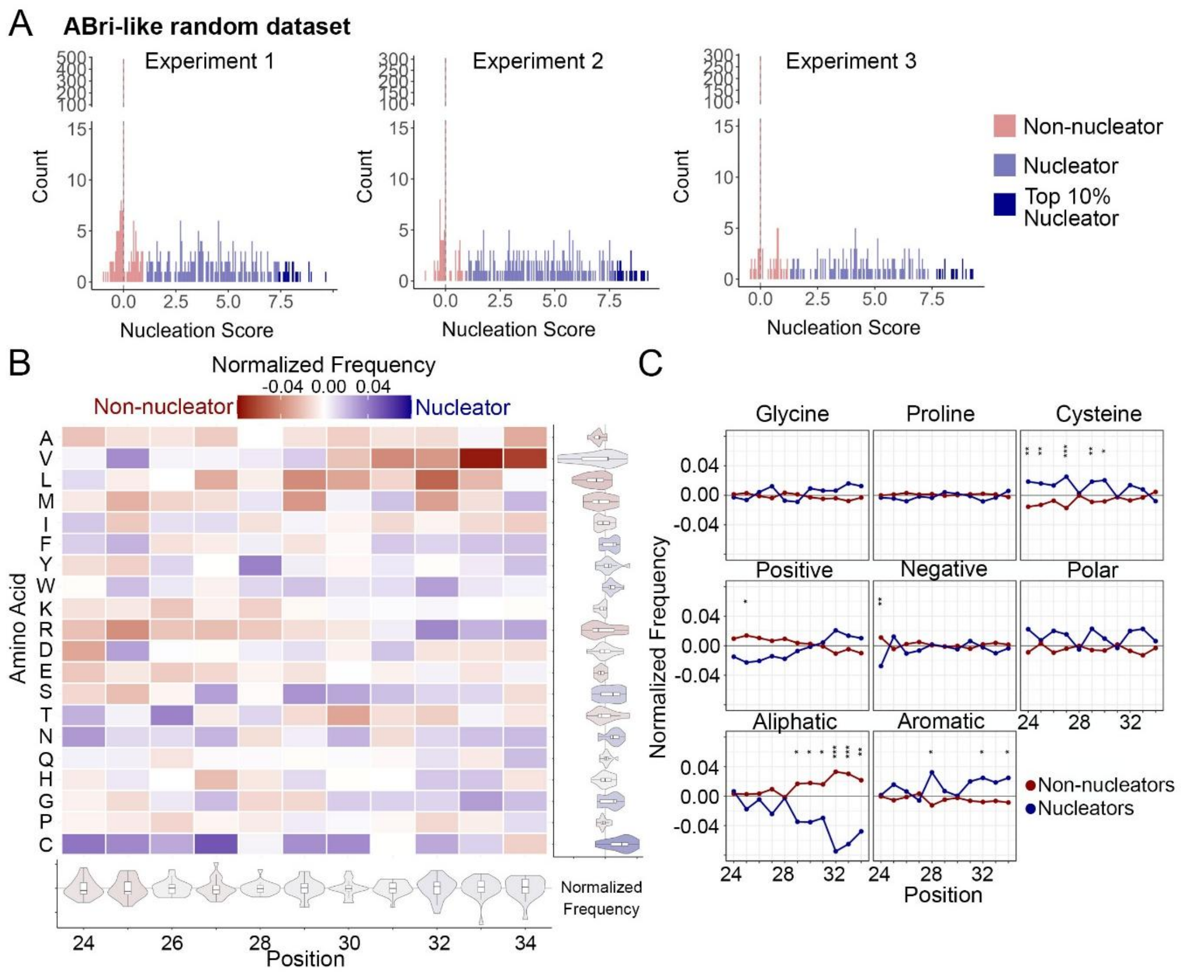

Supplementary Figure 8. ABri-like random dataset. A. Nucleation scores distribution of random sequences present in the three replicates. Variants are classified as non-nucleators (red), nucleators (blue) or top 10 nucleators (dark blue). **B.** Heatmap of normalized frequencies for ABri-like random extensions. The distribution of normalized frequencies for each position is summarized in the violin plots below the heatmap and the distribution of normalized frequencies for each mutation is summarized in the violin plots at the right-hand side of the heatmap. **C.** The position-specific differences in amino acid type frequencies across nucleating and non-nucleating sequences. Asterisks indicate marginal p-value (chi-square test). p<0.05: *; p<0.01: **; p<0.001: ***.

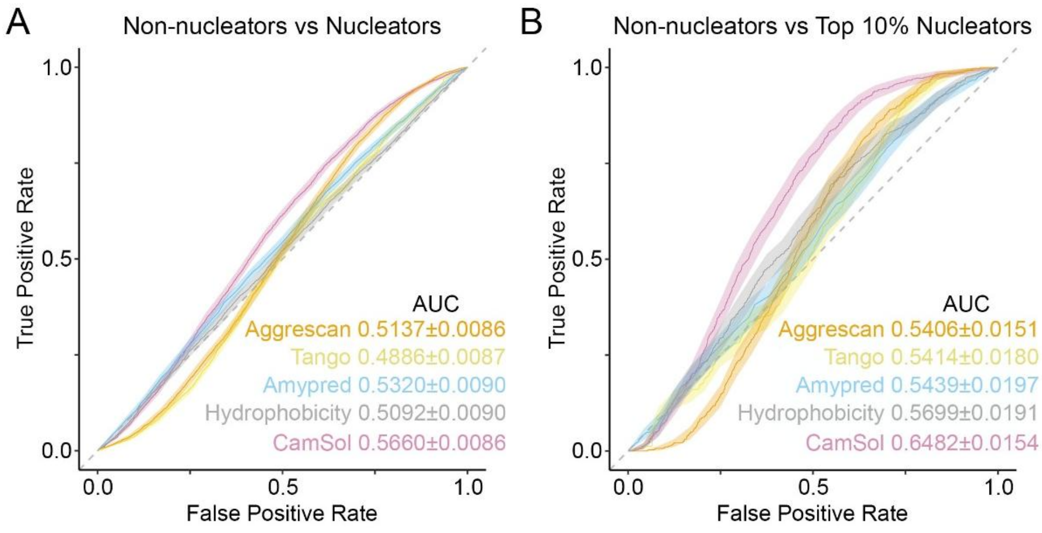

Supplementary Figure 9. Performance of amyloid predictors on Bri2 random extension dataset. ROC curves of state-of-the-art amyloid predictors on non-nucleators vs nucleators (**A**) and non-nucleators vs top 10% nucleators sequences (**B**). AUC and confidence interval is detailed for each of the predictors.

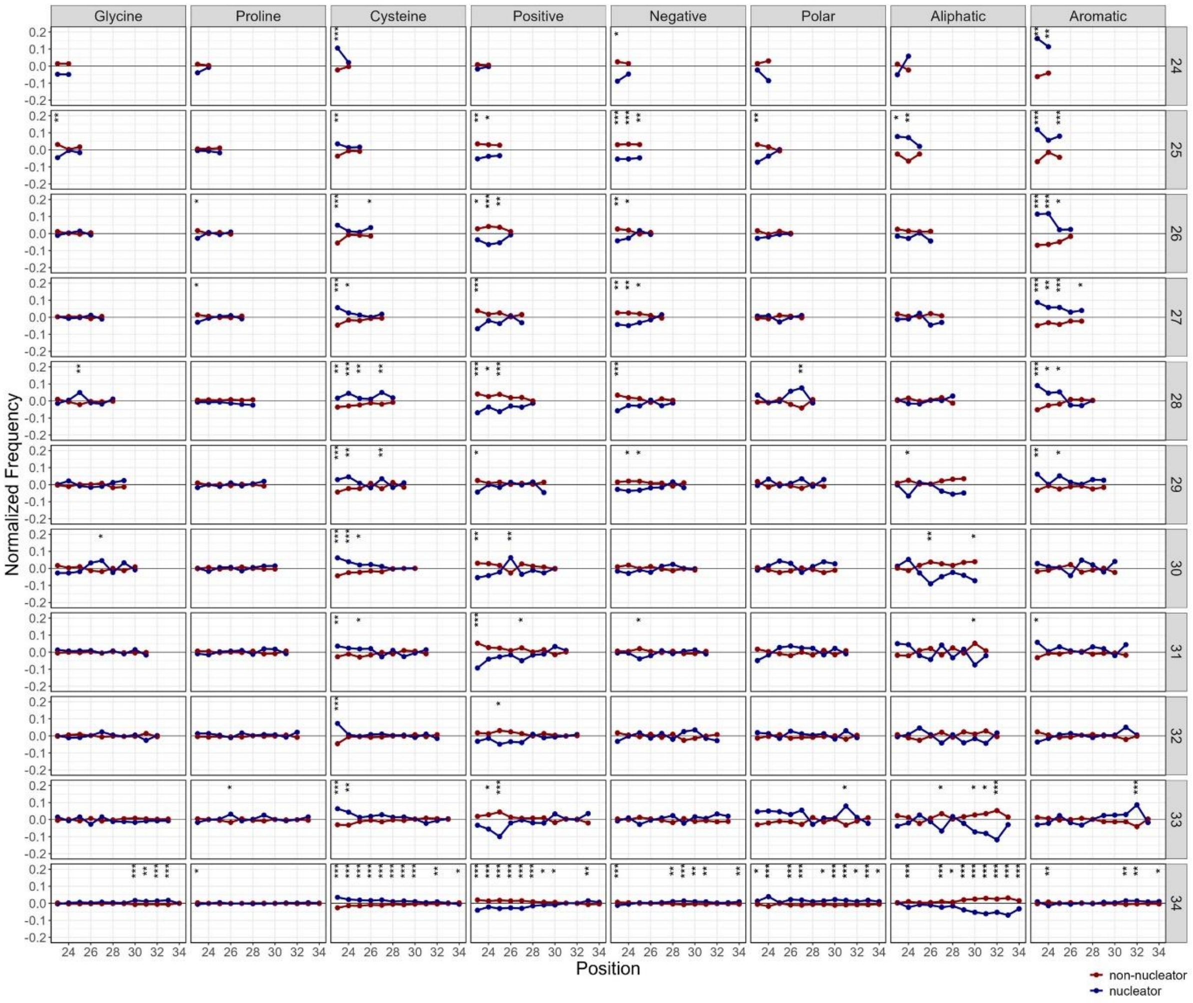

Supplementary Figure 10. Position-specific differences in amino acid type frequencies for truncated sequences across nucleating and non-nucleating sequences. Asterisks indicate marginal p-value (chi-square test). p<0.05: *; p<0.01: **; p<0.001: ***.
